# Endoscopic Grading as a Predictor to Develop Strictures in Corrosive Esophagitis in Children

**DOI:** 10.3390/jcm12041699

**Published:** 2023-02-20

**Authors:** Ioana Badiu Tisa, Lia Pepelea, Alexandru Pirvan, Iulia Lupan, Gabriel Samasca, Madalina Adriana Bordea

**Affiliations:** 1 Department of Mother and Child, Pediatric Clinic III, Iuliu Hatieganu University of Medicine and Pharmacy, 400217 Cluj-Napoca, Romania; 2 Emergency Hospital for Children, 400001 Cluj-Napoca, Romania; 3 Department of Molecular Science, Microbiology, Iuliu Hatieganu University of Medicine and Pharmacy, 400338 Cluj-Napoca, Romania; 4 Department of Mother and Child, Pediatric Clinic II, Iuliu Hatieganu University of Medicine and Pharmacy, 400177 Cluj-Napoca, Romania; 5 Department of Molecular Biology, Babes Bolyai University, 400371 Cluj-Napoca, Romania; 6 Department of Functional Biosciences, Immunology and Allergology, Iuliu Hatieganu University of Medicine and Pharmacy, 400162 Cluj-Napoca, Romania

**Keywords:** corrosive esophagitis, incidence, grading

## Abstract

Introduction. The incidence of corrosive esophagitis, also known as caustic esophagitis in children, is still increasing in developing countries, according to different clinical reports. Acids and alkalis are, in the same manner, involved in the pathogenesis of corrosive esophagitis in children. The aim of our study was to determine the incidence and endoscopic grading of corrosive esophagitis in a cohort of children from a developing country. Materials and methods. We performed a retrospective analysis of all pediatric patients who were admitted for corrosive ingestion at Pediatric Clinic II, Emergency Hospital for Children, Cluj-Napoca, over 10 years. Results. A total of 22 patients consisting of 13 (59.09%) girls and 9 boys (40.91%) were found in the present research. The majority of children lived in rural areas (69.2%). The results of laboratory tests were not well correlated with the degree of the injury. White blood cell counts over 20,000 cells/mm^3^, an increase in the C-reactive protein level and hypoalbuminemia were noticed only in three patients with strictures. The lesions were associated with *increased levels* of the *pro*-*inflammatory cytokines*, including interleukin (IL)-2, IL-5 and Interferon-gamma. Severe late complications such as strictures have been noticed in children with grade 3A injuries. The endoscopic dilation was done after the six months endoscopy. None of the patients treated with endoscopic dilation required surgical intervention for esophageal or pyloric perforation or dilation failure. The majority of complications (such as malnutrition) were noticed in children with grade 3A injuries. In consequence, prolonged hospitalization has been required. The second endoscopy (done six months after ingestion) revealed stricture as the most common late complication (n = 13, 60.60%: eight patients with grade 2B and five with grade 3A). Conclusion. There is a low incidence of corrosive esophagitis in children in our geographic area. Endoscopic grading is a predictor of late complications such as strictures. Grade 2B and 3A corrosive esophagitis are likely to develop strictures. It is crucial to avoid strictures and to prevent malnutrition.

## 1. Introduction

The incidence of corrosive esophagitis, also known as caustic esophagitis in children, is still increasing in developing countries, according to different clinical reports. This is due to the fact that prevention is lacking. Corrosive esophagitis occurs from the swallowing of different chemicals. Both alkalis and acids can induce corrosive esophagitis in children. It is more often caused by the ingestion of alkalines. This is due to their properties. Alkalines are usually colorless and relatively tasteless. In contrast, acidics have a pungent smell and a disagreeable taste. Corrosive esophagitis usually occurs from accidental or suicidal ingestion of chemical substances such as household disinfectants, bleaches or washing soda. A higher incidence is found in children below 10 years of age. Most of these caustic agents are consumed in a fluid state, and accidents usually take place indoors. In young patients, caustic ingestion is due to accidents at home and inadequate storage of caustic agents.

In the presence of relevant history, the clinical diagnosis is obvious. In the acute phase of the disease, patients present with intense oropharyngeal and chest pain associated with vomiting, excessive salivation and drooling. Haematemesis may also be present in some cases. Upper airway involvement leads to severe respiratory distress, stridor and hoarseness. Severe chest pain radiating to the back associated with episodes of fever and cough may suggest esophageal perforation. Epigastric pain or severe abdominal pain can occur in the presence of gastric injury. In the chronic phase, scarring and fibrosis lead to esophageal stricture, which presents as dysphagia, regurgitation, substernal discomfort, and recurrent aspiration. In contrast, gastric strictures present with vomiting, early satiety and weight loss. There is a high risk of malnutrition in children. Epiglottic involvement may lead to stridor, hoarseness and recurrent aspirations. Recurrent pulmonary infections are noted, especially in young patients. In the chronic stage, esophageal perforation may result in pneumomediastinum and mediastinitis, which may lead to the formation of a mediastinal abscess and death. The management of corrosive ingestion and its consequences remains an important medical challenge and public health issue. It is crucial to avoid strictures and to prevent malnutrition, especially in infants. In an emergency, it is useful to remove the soiled clothes, rinse the affected area and prevent vomiting and feeding. Prevention is very important for avoiding caustic ingestions in children. Information and education should be given specifically to the parents of toddlers; caustic products should be stored out of reach of children, and they should not be kept with food and drinks [1,2,3,4].

Early endoscopy (within 24–48 h after ingestion) is of great importance to detect the grade of the injury and, at the same time, for prognostic outcomes. The endoscopy (within 12–24 h following ingestion) permits careful assessment of anatomic derangements, serving as a valuable aide in decision-making in order to guide the need for further interventions. Delayed endoscopy (>48 h) should be avoided due to the increased risk of perforation as the result of tissue edema and inflammation. Imaging also has an important role to play. CT scan has a role in the diagnosis of some complications such as strictures. Severe forms of corrosive esophagitis are often characterized by hemorrhagic lesions, mediastinitis due to perforation, and a high risk of death. In contrast, stricture, one of the more serious late complications, may require months or years of dilatations or extensive surgical procedures for reconstruction. The incidence of esophageal carcinoma is also higher in patients with esophageal injury than in healthy people. Several studies suggest that only a particular type of lesion (caused by pesticide ingestion) is correlated with a high risk of esophageal carcinoma. Preexisting pesticide intoxication was associated with a 2.5-fold higher risk of esophageal cancer compared with the general population [5,6,7]. The pathogenesis of esophageal squamous cell carcinoma following caustic injury remains uncertain. The incidence of cancer in patients with corrosive strictures has been estimated to be around 6.2%. A history of caustic ingestion was present in 1% to 4% of patients diagnosed with esophageal carcinoma. The studies suggest that neither dilatation treatment nor esophageal bypass surgery can prevent the development of esophageal cancer. Epithelial dysplasia has been assumed to be a precancerous lesion. For alkaline ingestion, in particular, subsequent development of squamous cell carcinoma has been reported to occur approximately 40 years after the initial injury [4,8]. The damages produced by these chemicals are related to different mechanisms. Alkalis produce liquefaction necrosis. This kind of necrosis leads to the destruction of esophageal mucosa within a few minutes. Next days after the alkali consumption, mucosal injury is worsened by small vessel thrombosis, bacterial invasion, collagen deposition and ulcer production [8,9]. During this period, there is a high risk of perforation. Dysphagia and salivation are predictive of esophagitis, whereas abdominal pain, nausea and emesis are representative of gastric injury. Upper endoscopy is usually carried out during the first two days, and it can be safely carried out up to four days from the time of injury. The risk of perforation is higher in endoscopies performed later on, between 5 and 15 days. The healing process begins three weeks after ingestion. In contrast, acidic substances cause coagulation necrosis which may limit transmural injury due to the protective esophageal eschar formation. The stomach is not affected as gastric acid can neutralize alkaline agents. However, in cases of acidic substances, the esophagus can be spared while the stomach is severely injured. The esophageal protective eschar and lower surface tension allow acids to bypass the esophagus [8,9,10]. The injury depends on the concentration, quantity, type, and time of contact between chemical substances and the esophagus and pH value. Agents in the solid state are involved in mouth and pharynx lesions due to their quick attachment to those sites, while agents in the fluid state pass through them, causing important damage to the esophagus and stomach. Corrosive esophagitis affects in generally the middle and lower parts of the esophagus [8,10].

Endoscopic grading of corrosive esophagitis, using Zargar endoscopic classification, holds prognostic [9]. The grade of the injury, determined during the first endoscopic examination, is an important predictor for strictures. Endoscopic grades 1, 2 (2A) are considered “low-grade” injuries, whereas injuries falling into grades 2B, 3 and 4 are recognized as “high-grade” injuries. Chronic complications are not usually seen in cases of grade 1 and 2 injuries. The majority of grade 1 and 2A patients fully recovered. In contrast, most of the patients with grade 3 corrosive esophagitis are likely to develop strictures. Currently, there is no clearly established therapeutic protocol for corrosive esophagitis in children and adults. The utility of corticosteroids is controversial. The steroids are usually reserved for patients with symptoms involving the respiratory tract. Antibiotics are considered to only be administered in cases where there is evidence of perforation or infection. The time for the operation of esophageal replacement after a corrosive injury is still unknown. Emergency surgical exploration is only indicated if perforation or penetration is demonstrated. The most common organs used for esophageal replacement in patients after caustic injuries included: the stomach, jejunum, and colon. The colon is believed to be an ideal organ for replacement. When the hypopharynx is completely stenosed, a trans epiglottic approach is used. The treatment of children with esophageal strictures and involvement of the hypopharynx caused by caustic substance ingestion continues to be challenging [11,12,13]. Until this moment, there are no data regarding the incidence and endoscopic grading of corrosive esophagitis in our geographic area. The aim of our study is to determine these parameters in a cohort of children from Romania. It is well-known that the incidence of corrosive esophagitis in children is still increasing in developing countries, according to different clinical reports.

## 2. Materials and Methods

We performed a retrospective analysis of all pediatric patients who were admitted for corrosive ingestion at Pediatric Clinics II, Emergency Hospital for Children, Cluj-Napoca, over 10 years. A total of 22 children were diagnosed with corrosive esophagitis. All children undertook upper endoscopy within 24 h of their hospital admission. The esophageal injuries were graded using Zagar endoscopic classification [9,12,13] scale (Table 1).

The inclusion criteria were patients with positive anamnesis and specific symptoms of caustic ingestion, such as dysphagia, nausea, emesis, fever, or drooling of saliva. This study included only symptomatic patients.

Parents have been fully informed regarding the endoscopies. Parental consent regarding endoscopy was acquired for all participants. The exclusion criteria were children with a significant systemic inflammatory response. The endoscopies were performed by the same gastroenterology specialists. The caustic agent has been identified based on anamnesis and toxicological examination. The research was approved by the Ethical Committee of the Iuliu Hatieganu University of Medicine and Pharmacy Cluj-Napoca (187/2012—1 CD-ROM). Patients with grade 1 endoscopic findings were clinically observed and then discharged from the hospital with a 4-week follow-up. Proton pump inhibitors were prescribed for all patients, whereas corticosteroids and antibiotics were administered to all children with grade 2 and 3 injuries.

## 3. Results

A total of 22 patients consisting of 13 (59.09%) girls and 9 boys (40.91%) with a mean age of 7.98 years were included in our study. The majority of children lived in rural areas (69.2%). Accidental ingestion occurred in all our pediatric patients. Regarding the percentage distribution of the nature of ingested substances: 13 (59.09%) ingested alkaline agents (caustic soda—sodium hydroxide), 6 (27.27%) ingested acidics, while 3 (13.63%) patients ingested other corrosive agents. Endoscopic findings revealed grade 2 B injuries as the most frequent forms of caustic injury (n = 11, 50.00%), followed by grade 1 injuries (n = 6, 27.27%) and grade 3A injuries (n = 5, 22.73%) (Table 2).

**Figure 1:**
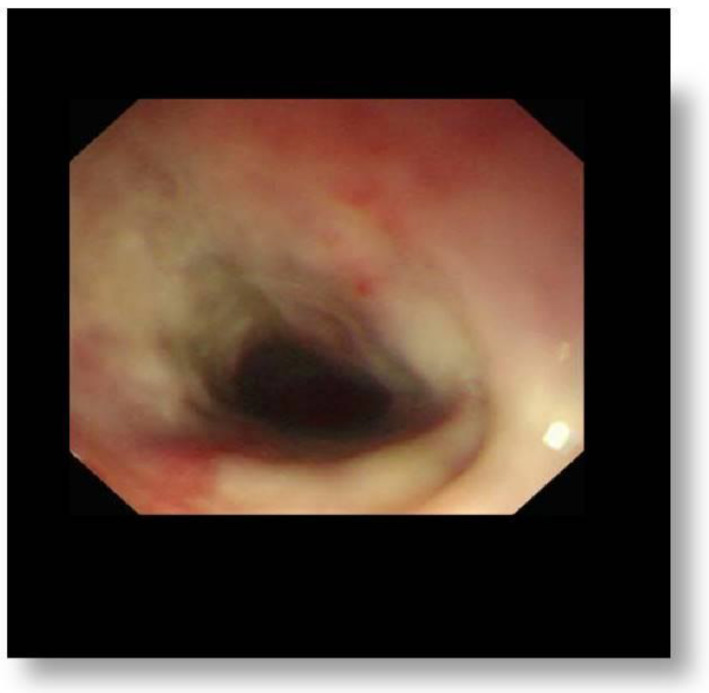
Stricture as the most common late complication.

The majority of complications (such as malnutrition) were noticed in children with grade 3A injuries. In consequence, prolonged hospitalization has been required. The second endoscopy (done six months after ingestion) revealed stricture as the most common late complication (n = 13, 60.60%: 8 patients with grade 2B (Figure 1) and 5 with grade 3A) (Table 3). None of our patients developed perforation.

Stricture treatments of patients with grade 3A corrosive esophagitis (n = 5) included endoscopic dilation (progressively time-spaced dilation-bougienage), medical treatment (proton pump inhibitors, corticosteroids, and antibiotics) and nasogastric feeding in two patients with a past history of gastroesophageal reflux disease. The endoscopic dilation was done after the six-month endoscopy. None of the patients treated with endoscopic dilation required surgical intervention for esophageal or pyloric perforation or dilation failure. The results of laboratory tests were not well correlated with the degree of the injury. White blood cell counts over 20,000 cells/mm^3^, an increase in the C-reactive protein level and hypoalbuminemia were noticed only in three patients with strictures. The lesions were associated with increased levels of the proinflammatory cytokines, including interleukin (IL)-2, IL-5 and Interferon-gamma.

Patients with grade 2B and 3A lesions require esophageal rest. These patients were managed with a combination of enteral or parenteral nutrition over a mean time of 21 days.

## 4. Discussion

There is a relatively high incidence of corrosive esophagitis in children, according to Fishman DS in an UpToDate article [14]. According to Mandana’s meta-analysis, annually, more than 40,000 cases of caustic ingestion in children are reported in England and Wales. It is still common in children in the United States, despite the decline of caustic ingestion, with an incidence of 15.8 cases in every 100,000 persons. From 2005 to 2006, 10% of the 51 children admitted to the Department of Pediatrics Children’s Hospitals from Iran had stenosis following ingestion of caustic agents [15]. Campos et al. revealed that most of the accidents occurred with children under three years of age (median: one year of age; interquartile interval: one to three years of age), at home (92.9%), and by ingestion (97.2%). Products involved were cleaning products with no caustic effects (38.9%), caustics (24.1%); hydrocarbons (19.3%); pesticides (16.6%), and other products (1.1%). Nineteen Portuguese children had to be hospitalized (17 patients were diagnosed with caustic esophagitis). No deaths occurred [16]. In Ghana, corrosive esophagitis in children under five years old is still common, with an incidence of 27 cases in every 100,000 persons [17]. From December 2005 to July 2008, 148 children were admitted for accidental caustic ingestion in Sierra Leone hospitals, according to Contini’s report. Overall, 126 patients were submitted to dilatation, with a mean of 4.9 (range: 1–23) procedures per child. A gastrostomy was done on 92 of 126 children (73%) [18]. Ngobese et al. managed 30 (60%) asymptomatic patients with no positive endoscopic and 20 symptomatic patients (75%) with oesophageal injuries (*p* = 0.01) in South Africa [19]. Similar results have been reported by DiNardo et al. in Italy. Their study reported 44 children with caustic ingestion. DiNardo suggested that endoscopic evaluation is mandatory in symptomatic patients to direct therapeutic [20]. In contrast, the ingestion of caustic substances remains a serious medical problem in Tunisian children. Rania et al. analyzed 1059 diagnostic procedures performed for caustic ingestion in children. The mean age of enrolled patients was 41.4 ± 31.9 months. The most frequently ingested caustic substance was household bleach, followed by caustic soda. Upper-Endoscopy showed severe esophageal and gastric lesions, respectively, in 122 (11.5%) and 56 (5.3%) cases. The occurrence of complications was significantly associated with the presence of severe gastric lesions at the initial endoscopic evaluation (21). These findings suggest that the incidence of corrosive esophagitis is still high in certain parts of the world. We reported only 22 cases over 10 years. The incidence is three times higher in under-developed and developing countries than in the Romanian population [4,8,18,21]. (Table 4). These controversies can be explained by the fact that the disease is underdiagnosed in our geographic area. The incidence of corrosive ingestion is probably largely unreported in our country. However, the incidence of asymptomatic corrosive esophagitis is not known. More data are needed to clarify the real incidence of corrosive esophagitis in Romania and different parts of the world.

Corrosive esophagitis is more often caused by the ingestion of alkalines. Alkalis (such as caustic soda) are found in commercially available household cleaners. Lesions are usually deeper and more penetrating with alkalis than with acidic compounds, but none of our patients developed perforation.

The preponderance of alkalis in reported pediatric data [4,8,9,10,18,19,20,21] can be explained very easily. Most accidental ingestion happens at home with chemicals found in different households. Children usually explore the environment as part of their normal development.

Caustic soda (sodium hydroxide) is a versatile alkali used in the manufacture of soap, detergents and different cleaners. For this reason, sodium hydroxide is easily accessible to them. In contrast, acidics (such as sulphuric acid) are handled mainly by car battery technicians. These exposures usually do not occur at home. Another explication is that alkalines are usually colorless and relatively tasteless. In contrast, Martins O Thomas et al. [22] revealed, in a case series of 22 individuals with corrosive esophagitis, that 55.1% of them ingested acids (battery water) while only 35.9% ingested alkalis, mainly caustic soda. Our data illustrate that the incidence of corrosive esophagitis caused by the ingestion of alkalines is higher than the incidence of corrosive esophagitis caused by acidics. These results are in line with most of the previous studies.

We conclude that the incidence rates of the disease were greater in girls than boys, and ingestion was accidental among our study population. According to different studies, corrosive esophagitis is common in male patients, and the ingestion is mostly accidental. We also report significant exposure among children living in rural areas. The higher prevalence among rural areas might be attributed to the more accessibility of concentrated caustic products for utilization for agricultural purposes. Our results were partially comparable to previous studies in which the majority of the populations involved were males [9,10,22,23].

Caustic substance ingestion produces mucosal damage and leads to excessive inflammatory cytokines in peripheral blood. This is in line with others data [24,25].

Our study showed that grade 2B injuries were the most common caustic injury. Our findings are in opposition to other clinical trials in adult and children populations in which cases were most commonly regarded as grade 2A corrosive esophagitis [3,8,10]. The occurrence of severe strictures during the second control endoscopy was observed in patients with grade 2B injuries, followed by grade 3A injuries, after initial therapy. Previous studies suggested that only grade 3A is predisposed to develop stenosis. These findings are in contrast with previous data. Probably early bougienage made the difference.

Strictures were more frequent in patients with corrosive esophagitis caused by ingestion of alkalines. The esophageal protective eschar and lower surface tension that allow acids to bypass the esophagus may explain the higher incidence of esophageal strictures after alkaline ingestion [3,8,10,22,23]. Corrosive esophagitis, in the same manner as other esophageal diseases [26,27,28,29] and their sequels constitute a medical challenge nowadays.

A systematic review and meta-analysis performed by Katibe et al. of 763 publications from which 244 patients were included in randomized clinical trials concluded that there was no benefit of corticosteroids and antibiotics administration in preventing the development of esophageal stricture formation. The utility of corticosteroids is controversial. The study did not find any benefit of steroid administration in terms of stricture prevention. The steroids are usually reserved for patients with symptoms involving the respiratory tract. Antibiotics are considered to only be administered in cases where there is evidence of perforation or infection. The administration of broad-spectrum antibiotics is usually mainly advised if corticosteroids are initiated. However, it can be used as an adjunct to steroids, which are usually found not to be of value for first and third-degree esophageal burns, whereas some benefit is expected in second-degree injury. Prophylactic use is not recommended. To date, the efficacy of proton-pump inhibitors and H_2_ blockers in minimizing esophageal injury by suppressing acid reflux has not been proven [30,31]. Recent studies concluded that topical mitomycin-C administration could be recommended to reduce the number of dilation procedures required in patients with esophageal stricture following corrosive chemical ingestion. Mytomycin-C is a cytostatic agent with severe side effects. Mytomycin-C is anthracycline derived from Streptomyces anti-fibrotic agent that inhibits fibroblast proliferation and decreases scar production. Application of topical mitomycin C with endoscopic dilations in caustic esophageal strictures was more effective in dysphagia resolution than endoscopic therapy alone in the pediatric population. Moreover, topical mitomycin C application also reduced the number of dilation sessions needed to alleviate dysphagia without rising morbidity. Data are needed on the effect of anti-fibrotic mitomycin-C used topically to prevent post-ingestion fibrosis, especially in young patients. Local application of mytomycin-C is a therapeutic option for the treatment of refractory esophageal strictures in children and adults [31,32,33].

## 5. Conclusions

The incidence of corrosive ingestion in symptomatic patients requiring admission decreased in developing countries such as Romania (only 22 patients over 10 years). The low incidence rate is due to the fact that parents are educated to keep household corrosives safely away from children. However, the incidence of asymptomatic corrosive esophagitis is not known. More data are needed to clarify the real incidence of corrosive esophagitis in Romania and different parts of the world. The disease is more often caused by the ingestion of alkalines. Our research revealed grade 2B injuries as the most frequent form of caustic injury. Our study demonstrated that grades 2B and 3A are likely to develop strictures. None of our patients developed perforation, including those with alkalis. The incidence of esophageal strictures is higher in patients with corrosive esophagitis caused by ingestion of alkalines than in cases of corrosive esophagitis caused by acidic agents. It is crucial to avoid strictures and to prevent malnutrition, including in patients with grade 2B corrosive esophagitis since the treatment for the complication is very expensive. Thus, early bougienage helps to decrease the development of esophageal strictures. Regarding the risk for esophageal carcinoma, further studies are needed.

## Figures and Tables

**Table 1:** Modified Zargar’s endoscopic classification scale [6,9,12].

Grade 0	Normal examination
Grade 1	Edema and hyperemia of the mucosa
Grade 2A	Superficial ulceration, erosions, friability, exudates, hemorrhages, whitish membranes
Grade 2B	Grade 2A plus deep discrete or circumferential ulcerations
Grade 3A	Small scattered areas of multiple ulcerations and areas of necrosis with brown-black or greyish discoloration
Grade 3B	Extensive necrosis

**Table 2:** Endoscopic quantification.

Corrosive Esophagitis	No. (n)	Percentage (%)
Grade 1	6.00	27.27
Grade 2B	11.00	50.00
Grade 3A	5.00	22.73

**Table 3:** The six months endoscopic findings/incidence of esophageal stricture.

Corrosive Esophagitis	No.	Grade 1 No. (%)	Grade 2B No. (%)	Grade 3B No. (%)	Stricture
Grade 1	6 (100)	6 (54.55)	0 (0)	0 (0)	0 (100)
Grade 2B	11 (100)	0 (0)	11 (27.27)	0 (0)	8 (72.72)
Grade 3A	5 (100)	0 (0)	0 (0)	5 (40)	5 (100)

**Table 4:** Incidence of corrosive esophagitis according to different reports.

Crt.	Country/Region	Number of Cases
1	England and Wales	4/100,000 persons /year
	USA	15.8/100,000 persons /year
	Iran	51/100,000 persons /year
	Portugal I	19/100,000 persons /year
	Ghana	27/100,000 persons /year
	Sierra-Leone	126/100,000 persons /year
	South-Africa	50/100,000 persons /year
	Italy	44/100,000 persons /year
	Tunisia	105/100,000 persons /year

## Data Availability

Not applicable.
